# Temporal dimensions in marine spatial closures

**DOI:** 10.1017/cft.2026.10034

**Published:** 2026-06-08

**Authors:** Erendira Aceves-Bueno, Matthew Lauer, Emilie Lindkvist, Olivia K. Isbell, Bonnie Basnett, Yutian Fang, Liliana Sierra Castillo, Amy Hudson Weaver, Stuart Fulton, Jean Wencélius, Steven D. Gaines, Anastasia C.E. Quintana

**Affiliations:** 1 School of Marine and Environmental Affairs, University of Washington, USA; 2 Department of Anthropology, San Diego State University Department of Anthropology, USA; 3 Stockholm University Stockholm Resilience Centre, Sweden; 4 Bren School of Environmental Science & Management, University of California Santa Barbara, USA; 5 Smithsonian Tropical Research Institute, Panama; 6 Independent Consultant, Mexico; 7 Comunidad y Biodiversidad AC, Mexico; 8 PSL Université Paris: EPHE-UPVD-CNRS, UAR3278 CRIOBE, Moorea 98728, French Polynesia

**Keywords:** adaptive management, community-based conservation, fisheries co-management, marine protected areas, marine spatial planning

## Abstract

The permanent closure of large marine areas is often regarded as an effective strategy for conserving marine biodiversity. However, implementing large permanent closures can be difficult in many small-scale fisheries systems, where communities depend heavily on marine resources for their livelihoods and food security, and where issues of equity and access must be carefully considered. Around the world, many coastal communities instead rely on *temporary closures* – areas that open and close over time – to balance ecological recovery with ongoing use. Despite their prevalence and potential, these dynamic approaches remain conceptually underdeveloped compared to permanent marine-protected areas. This article calls for a reevaluation of marine management’s prevailing focus on space by bringing *time* to the forefront of analysis. We introduce a framework that distinguishes two key temporal dimensions – duration and cyclicity – to clarify how different temporal designs shape ecological, social and governance outcomes. Within this framework, cyclicity captures how closures and openings alternate over time, including the relative balance between periods of access and closure (*i.e.*, the access ratio). Drawing on diverse examples where temporary management is implemented, this framework lays the groundwork for developing more formal theory and comparative evidence about how temporal strategies can align ecological recovery with livelihood dynamics in resource-dependent systems.

## Abstracto

En teoría, el cierre permanente de grandes áreas marinas es la vía más efectiva para conservar los recursos marinos. Sin embargo, tales medidas rara vez son factibles en regiones donde las comunidades costeras dependen directamente de estos recursos para su subsistencia y bienestar. En todo el mundo, muchas comunidades costeras recurren en cambio a cierres temporales—áreas que se abren y cierran a lo largo del tiempo—para equilibrar la recuperación ecológica con el uso continuo. A pesar de su prevalencia y potencial, estos enfoques dinámicos siguen estando conceptualmente poco desarrollados en comparación con las áreas marinas protegidas (AMP) permanentes. Este artículo propone una reevaluación del enfoque predominante de la conservación marina en el espacio, situando el tiempo en el centro del análisis. Presentamos un marco que distingue tres dimensiones temporales clave—duración y ciclicidad —para aclarar cómo los diferentes diseños temporales influyen no solo en los resultados ecológicos, sino también en los sociales y de gobernanza. Basándose en diversos ejemplos donde la gestión temporal prospera, este marco proporciona una base para la investigación comparativa sobre la dinámica socioecológica de los cierres marinos y mejora la comprensión de cómo la variación temporal puede contribuir a una conservación más adaptativa y sensible al contexto.

## Impact statements

Efforts to conserve the ocean often emphasize permanently protected areas, while many coastal communities also use time itself as a tool for stewardship. Around the world, fishers temporarily close and reopen fishing grounds to allow ecosystems to recover while sustaining their livelihoods. Despite their prevalence, scientific attention to these *temporary closures* has been limited – and existing studies have largely focused on their ecological effects. This overlooks the fact that such practices are often deeply rooted in social relations, cultural traditions and local governance systems – features that can enhance the long-term sustainability of fisheries management. This study introduces a framework that helps researchers analyze the temporal dimensions of these systems – how long closures last and how access is distributed over time – revealing how social and ecological processes interact through time. By bringing these dimensions together, the framework expands the foundation for comparative research and fosters a more complete understanding of the diverse ways people and communities care for the ocean.

## Introduction

The ocean is increasingly managed through spatially explicit policy tools such as marine spatial planning and marine-protected areas (MPAs). MPAs are widely considered an effective management tool for preventing the overexploitation of marine resources (Green et al., 2009; Gaines et al., 2010; Sala et al., 2021). These closures are designated areas where fishing and other activities are restricted or prohibited and aim to protect sensitive habitats, preserve biodiversity and allow fish populations to recover from overfishing. Several studies have revealed evidence of the effectiveness of large permanent fully protected areas for marine biodiversity protection, where restrictions on fishing and other activities are banned in perpetuity (McLeod et al., 2009; Grorud-Colvert et al., 2021; Sala et al., 2021). Large permanent fully protected areas have gained further support with the rise of the “30×30” initiative, where the goal is to protect at least 30% of the world’s oceans by the year 2030 (Pike et al., 2024; Robinson et al., 2024; Villasenor-Derbez et al., 2024). Although there is no consensus within the conservation community about what level of protection this target should entail (Stephenson et al., 2025; Henneker et al., 2026), the growing recognition of the importance of marine management and the need to restore and preserve marine ecosystems have led to increased advocacy for permanent closures as a means to achieve sustainable fisheries and safeguard marine biodiversity (Viana et al., 2024).

However, in regions where livelihoods and food security depend directly on marine resources, the permanent closure of large fishing areas is often impractical and the effectiveness of MPAs has been the subject of considerable debate (De Santo, 2013; Rife et al., 2013; Hilborn, 2018; Hilborn et al., 2022). MPAs also commonly operate better when there is a well-funded state apparatus or a non-governmental organization that can enforce the area, but in many coastal areas where these well-funded systems do not exist, MPAs can become “paper parks” existing only on paper with low compliance, highlighting the broader challenge of ensuring that management initiatives persist and remain effective over time (Rife et al., 2013; Pienkowski et al., 2026). The lack of local buy-in with top-down imposed MPAs can also lead to issues of justice and equity (De Santo, 2013; Bennett et al., 2021; Grorud-Colvert et al., 2021).

To address the challenges associated with MPAs, efforts have focused on both governance and spatial design. In addition to promoting more inclusive governance approaches, such as adaptive co-management (Bown et al., 2013), experts have worked extensively on refining the spatial configuration of MPAs. Optimizing elements like size, spacing, shape and connectivity is intended to enhance ecological resilience, reduce vulnerability to poaching and increase stakeholder support by securing fisheries benefits (Green et al., 2009; McLeod et al., 2009; Gaines et al., 2010). These approaches are often implemented alongside regulations on fishing activities (*e.g.*, gear restrictions or species-specific rules), underscoring that spatial design is rarely independent from the types of activities being managed.

Crucially, most of these efforts do not engage in a comprehensive exploration of temporal aspects of protection. Area-based fisheries management and conservation measures are often conceptualized along three interrelated dimensions: space (where management occurs), time (when restrictions apply) and activity (what human actions are regulated, such as gear types or fishing practices). Existing ABMT typologies provide a useful foundation for organizing these dimensions across marine management systems (Hilborn et al., 2022), but they generally treat temporal aspects as categorical rather than structural, limiting systematic comparison across different temporal designs. The literature has also historically emphasized the regulation of activities through gear restrictions, quotas and effort controls and the spatial design of interventions such as MPAs and marine spatial planning. In contrast, far less attention has been given to the temporal dimension of protection. Instead, permanency is often positioned as the standard for achieving management goals such as the 30x30 initiative, neglecting the broader diversity of temporary management approaches (Dudley, 2008; Wenzel et al., 2020; Pike et al., 2024). This tendency includes Other Effective Area-based Conservation Measures (OECMs) which must be “ongoing and for the long-term” – a definition that remains open to interpretation and may, in some contexts, include non-permanent measures while still implying extended protection (IUCN WCPA Task Force on OECMs, 2019). Together, these approaches reflect a broader paradigm in global marine management that emphasizes fixed spatial protections while treating time as static or secondary.

Yet, temporal management is neither new nor marginal. In the Global North, fisheries management has long relied on seasonal closures, rotational harvests and real-time restrictions to regulate fishing effort and protect spawning periods. In many parts of the Global South, however, communities have long practiced non-permanent closures, whether spatially fixed or shifting, rooted in local stewardship traditions, often serving broader socio-ecological and cultural objectives. These practices represent a rich but underexplored dimension of temporary marine management (Johannes, 1978).

Research on these tools has focused primarily on ecological outcomes, examining both their benefits and potential downsides (Hart, 2003; Micheli et al., 2012; Lewison et al., 2015; Little et al., 2015; Munguía-Vega et al., 2015; Plagányi et al., 2015; Chen and Hastings, 2023). Importantly, much of the existing literature examines temporal closures primarily as fisheries management instruments – typically seasonal or effort-control measures designed to protect spawning periods or regulate harvest. While these approaches are widespread and well documented, they represent only one form of temporal marine governance. In many parts of the world, communities also implement temporary closures that pursue broader socio-ecological goals, including biodiversity conservation, ecosystem stewardship and the maintenance of cultural traditions. These diverse examples of temporal fisheries management highlight the importance of studying not only ecological outcomes but also the socio-cultural and political dimensions of temporary marine management.

Non-permanent spatial marine resource management has a long history across Pacific Island regions. Across the Indo-Pacific, communities have widely implemented periodically harvested closures, a form of rotational or temporary closure used to manage coral reef fisheries and support multiple ecological and socio-economic (Bartlett et al., 2009; Carvalho et al., 2019a; Cinner et al., 2006; Cohen et al., 2013; Cohen and Foale, 2013; Jupiter et al., 2017. These systems are particularly prevalent in Melanesia, where customary marine tenure and traditional ecological knowledge enable communities to adaptively manage reef resources through periodic closures and pulse harvesting strategies (Goetze et al., 2018). Specific local governance systems illustrate this broader pattern. In Papua New Guinea, communities with strong tenure rights implement periodic closures that can generate ecological and fisheries benefits under certain conditions (Cinner et al., 2006; Bartlett et al., 2009; Cohen et al., 2013; Cohen and Foale, 2013; Jupiter et al., 2017). In French Polynesia, communities are implementing temporary management forms called rāhui – closures historically established for ritual or social purposes that have increasingly been adapted for conservation and fisheries management (Bambridge et al., 2019; Oliver, 2019; Filous et al., 2021). Contemporary *rāhui* vary in objectives and design, but typically involve periodic openings during a multi-year closure period, after which they may expire or be extended (Bambridge et al., 2019; Oliver, 2019).

Similarly, in the Western Indian Ocean, temporary octopus closures have been reintroduced across several communities (Oliver et al., 2015; Silas et al., 2022; Dudayev et al., 2023). These closures last for defined periods each year, temporarily banning octopus fishing and culminating in coordinated, celebratory harvests. Their predictable annual timing and strong local participation enhance food security, livelihoods and community cohesion. Some communities also adjust the location or boundaries based on socio-ecological feedbacks (Wosu, 2019; Drury O’Neill et al., 2024).

Another example is found in Mexico, where “Zonas de Refugio” (fish refuges, in English) are fixed-term closures, typically lasting 5 years, during which fishing can be entirely prohibited. Initiated by small-scale fishers and non-governmental organizations following a 2007 fisheries law reform, they are intended to allow stock recovery (Quintana and Basurto, 2021). Fixed-term closures are also used elsewhere; for instance, in New Zealand, they can last for up to 2 years (Gnanalingam and Hepburn, 2015).

Despite their global relevance, temporary marine closures and the temporal dimensions of marine management they represent, have received far less attention in the literature than the spatial dimensions associated with permanent closures. A key challenge is the conceptual ambiguity of “temporary,” which groups diverse practices – seasonal bans, event-triggered closures, rotational systems and traditional time-bound regimes – under a single label. This broad categorization obscures how temporal design shapes not only ecological but also governance and social outcomes. Disentangling these effects requires explicit consideration of how temporal dynamics interact with spatial configurations and the regulation of activities, which are often deeply intertwined in practice. To address this gap, we introduce a conceptual framework focused on key temporal dimensions of marine closures. The framework clarifies distinctions between permanent and temporary systems, refines the language used to describe temporal strategies and helps identify avenues for future research. While our primary contribution is to advance understanding about temporal dimensions, we explicitly situate this framework within the broader space–time–activity paradigm and highlight the need for future empirical work to account for variation in regulated activities when evaluating the outcomes of temporary closures. Our goal is not to present temporary closures as a panacea, but to provide a framework for more precisely evaluating how spatial and temporal configurations shape marine management in resource-dependent communities, including how these approaches can complement other management strategies.

## Key temporal characteristics of marine closures

**Figure 1.**
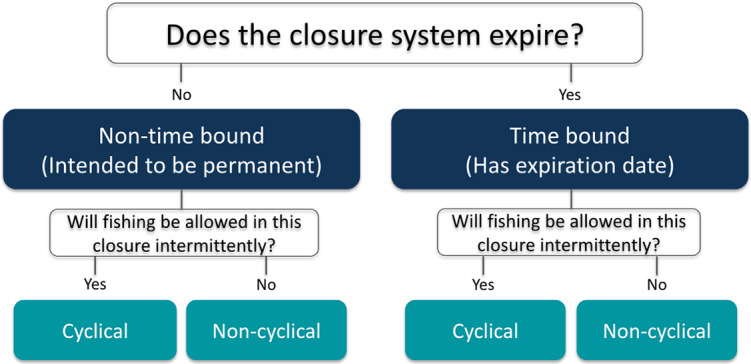
Temporal classification of marine closures based on system duration and cyclicity. This framework distinguishes closures by whether they are time-bound (designed to expire or be reassessed after a set period) or non-time-bound (intended to persist indefinitely), and by whether they follow a cyclical pattern (with alternating open and closed periods) or are non-cyclical (remaining consistently closed or open). Cyclical closures can also be characterized by their access ratio – the proportion of time that resources are accessible to users – which reflects the intended balance between ecological protection and stakeholder use (Figure 2). Figure 1. long description.

The term “temporary closures” is an umbrella concept, grouping together distinct management approaches and hindering both research and practice. To address this ambiguity, we propose a simple, yet powerful framework that distinguishes two key temporal characteristics: *system duration* and *cyclicity* (Figure 1).

*System duration* refers to whether closures are intended to be time-bound, with a defined expiration, or non-time-bound, designed to persist indefinitely. Time-bound closures, such as Mexico’s 5-year fish refuges, may be extended following evaluation, allowing for adaptive redesign based on outcomes. This deliberate, time-limited structure enables flexibility and iterative learning in management (Quintana and Basurto, 2021). System duration strategies are underpinned by enduring institutions – formal or informal – that provide the governance foundation for their implementation (Ostrom’s “constitutional rules”) (Ostrom 2012). For example, Mexico’s fish refuges are supported by the 2007 General Law of Sustainable Fisheries and Aquaculture. Thus, while institutions persist, our definition of *time-bound* refers specifically to operational rules – the in-practice arrangements – rather than the underlying legal or normative structures.

*Cyclicity* is a second key temporal dimension we have identified that describes crucial temporal characteristics of closures. Cyclical closure strategies involve a predetermined or *ad hoc* sequence of opening and closing periods of harvesting, whereas non-cyclical closures are designed to be closed indefinitely and not reopen for harvesting once established. The most well-known cyclical closures are seasonal closures, where a management area is designed to alternate between states of protection and access. A cyclical management system introduces periodic access to resources for resource users and can be designed to help align ecological and social rhythms.

**Figure 2.**
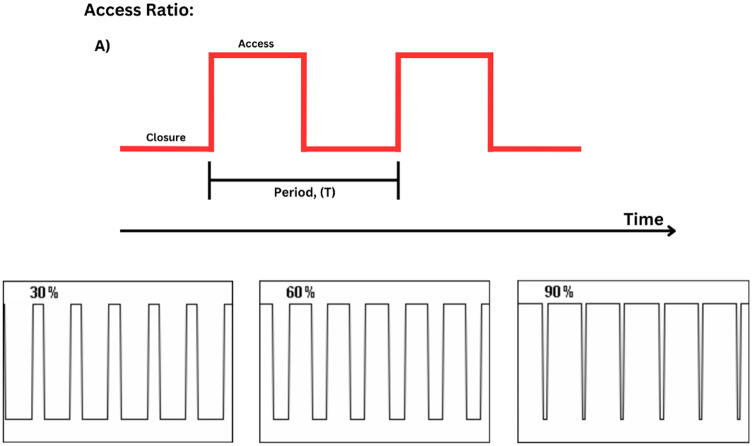
Access ratio in cyclical marine closures. This figure illustrates the concept of the access ratio, which describes the temporal dynamics of cyclical closures. Panel A defines the components of the access ratio, including the durations of open and closed periods within a time frame (T). Panels B–D provide examples of closures with access ratios of 30%, 60% and 90%, respectively, demonstrating varying proportions of fishing access within a given period. While the access ratio is inspired by the concept of duty cycles in engineering, it does not require strictly repetitive patterns as those shown in these examples; the lengths of open and closed periods can vary dynamically across time frames, potentially reflecting adaptive management strategies. Figure 2. long description.

Cyclical closures can be further assessed through their *access ratio* – the proportion of time resources are available to users (Figure 2). This ratio shapes social and ecological outcomes: well-timed closures can support species recovery and fishery sustainability, while extended access may lead to overfishing, and limited access may reduce community support. In our framework, we emphasize access ratio primarily in the context of cyclical closures, where the proportion of open and closed periods is intentionally designed to align ecological and social objectives. Nonetheless, access ratio can also be applied more broadly to any temporary closure, including event-triggered or non-cyclical systems, to describe the realized proportion of time that resources are accessible. In these cases, the ratio emerges from management responses rather than deliberate design, but it still provides a useful comparative measure across diverse closure strategies.

**Figure 3.**
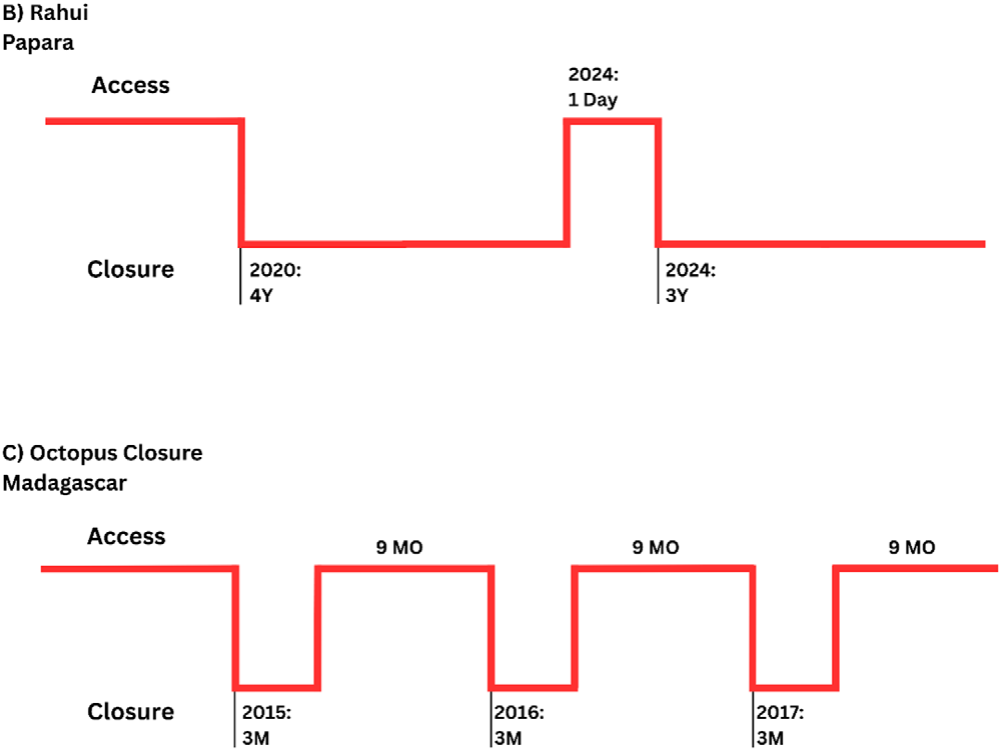
Representation of the access ratio (red line) applied to select case studies. Access periods represent the length of time that communities have access to fishing. Closure periods represent the length of time communities cannot access the fishery. To enhance pattern recognition, the duration of cycles of each example is not depicted on the same timescale; instead, the specific durations are stated in years (Y), months (MO) and days. The figure highlights differences in access-to-closure ratios for the following cases: a) Papara *rāhui* and b) octopus closures in Madagascar. Figure 3. long description.

*System duration, cyclicity* and *access ratio* can help distinguish key temporal characteristics of management systems lumped together as “temporary closures” (Figure 3). For example, in French Polynesia, *rāhui* practices vary widely: On Maiao and Rapa Iti, the *rāhui* are cyclical, non-time-bound with eventual shifting zones. In Teahupo’o, the *rāhui* was initially designed as time-bound but non-cyclical, spatially fixed for a period of 3 years. In Papara and Tautira, *rāhui* are also time-bound but allow for cyclical openings within that period. While in the Western Indian Ocean, octopus closures are designed as non-time-bound, and cyclical, typically involving months-long closures followed by short harvest periods. Conventional trigger closures can be characterized by our framework as non-cyclical, time-bound measures activated in response to management or conservation concerns (Gullestad et al., 2015; Little et al., 2015; Bisack and Magnusson, 2021).

Our framework positions time as a fundamental design axis, offering precise vocabulary to uncover the nuanced trade-offs obscured by the broad “temporary” label.

## Future research directions

By highlighting key temporal attributes of marine closures, our framework reveals new research directions and underscores how variations in *system duration* and *cyclicity* influence socio-ecological trade-offs and management outcomes. We have identified four focal areas of research to guide and motivate future inquiry. These include (a) ecological and fisheries sustainability; (b) governance, equity and participation; (c) adaptability and learning; and (d) economic resilience (Table 1).

**Table 1.** *Major* research areas and associated questions examining the ecological, social, governance and economic dimensions of area-based marine management through a temporal lens, explicitly incorporating activity (*e.g.*, gear, effort and targeting behavior) and additional mediating variables (*e.g.*, governance capacity, market dynamics and ecological heterogeneity) that condition outcomes across systems Table 1. long description.

Potential research questions	Hypotheses	Variables of interest
a. Fisheries sustainability		
How does the synchronization of access regimes with species’ ecological traits (*e.g.*, mobility, recovery time) influence fisheries outcomes and fisher perceptions?	Synchronizing access regimes with species’ ecological traits enhances fisheries outcomes and strengthens fisher engagement by providing timely and visible benefits.	Temporal design: duration; cyclicity Species life-history traits: mobility; recovery rate Activity (key mediating variable): gear selectivity; effort allocation; targeting behavior Spatial structure: distribution of stocks/habitats Social outcomes: fisher perceptions Additional mediating variables: governance capacity; enforcement strength; market dynamics; ecological heterogeneity
How do different access ratios interact with gear selectivity, habitat sensitivity and species life histories to determine whether temporary closures generate ecosystem-wide benefits beyond stock-specific recovery?	Variations in access ratios, gear selectivity, habitat sensitivity and species life histories significantly influence whether temporary closures yield ecosystem-wide benefits beyond stock-specific recovery.	Access ratio Activity (key mediating variable): gear selectivity; effort allocation; targeting behavior influencing response to access design Ecological structure: habitat sensitivity; species life histories Spatial structure: habitat configuration Additional mediating variables: governance enforcement capacity; market conditions; ecological heterogeneity
How does temporal access to fisheries affect recovery and trophic interactions across different levels of marine food webs?	Periodic human access to marine closures can mimic the ecological role of top predators by regulating prey populations, but it may also limit the recovery of high trophic level species that are slower to rebound. Therefore, the effects of closure timing on ecological recovery are predicted to vary across trophic levels, reflecting the dual role of humans as resource users and functional predators.	Temporal design: duration; cyclicity; access ratio Ecological structure: trophic level structure; species recovery rates Activity (key mediating variable): fishing pressure (effort; gear type; targeting intensity) Spatial structure: food-web structure Additional mediating variables: ecosystem productivity; climate variability; governance enforcement strength
b. Governance, equity and participation		
How do the duration of closures and the proportion of time resources are accessible (access ratios) influence community perceptions of fairness, equity and governance legitimacy?	Closures that balance sufficient protection for resource sustainability with predictable and meaningful access opportunities are expected to enhance perceptions of governance legitimacy. Communities are likely to perceive rules as fair and equitable when access ratios allow sustainable harvests of key targeted species and when allocation rules are transparent and inclusive. Conversely, closures with very restrictive access or poorly communicated rules may undermine perceptions of fairness and equity, even if ecological outcomes are positive.	Temporal design: duration; access ratio; cyclicity Governance design: rule transparency Activity (key mediating variable): gear restrictions; effort rules shaping access outcomes Spatial design: allocation of access Social outcomes: equity Additional mediating variables: enforcement capacity; institutional trust; market dependence
How do time-bound and cyclical marine closures influence local agency?	Time-bound and cyclical closures enhance local agency by requiring communities to actively monitor resources and make decisions about openings and extensions. Unlike permanent closures, which can lead to user disengagement, these temporary management strategies provide regular feedback on ecological outcomes, enabling adaptive responses. Consequently, communities involved in managing time-bound or cyclical closures are expected to demonstrate higher participation, more informed decision-making and greater capacity to adjust management practices based on observed ecological changes.	Temporal design: duration; cyclicity; access ratio Governance process: participatory governance structure Activity (key mediating variable): gear/effort regulation shaping agency outcomes Spatial design: exclusivity Additional mediating variables: governance history; institutional capacity; community cohesion
How can the design of time-bound and cyclical marine closures support the restoration or revitalization of pre-colonial, community-based governance systems?	Time-bound and cyclical closures, by reflecting flexible, context-specific rules for resource use, can reinforce or revive pre-colonial governance practices that emphasized local stewardship and communal decision-making. By aligning closure timing with ecological cycles and community needs, these management strategies may strengthen local institutions, encourage participatory rule-making and restore customary norms.	Temporal design: duration; cyclicity; access ratio Institutional context: customary tenure systems; governance traditions Activity (key mediating variable): rules on gear use; effort constraints shaping customary alignment Spatial structure: territorial governance boundaries Additional mediating variables: cultural continuity; institutional legitimacy; enforcement capacity
How does participatory decision-making in reopening or extending marine closures influence cooperation, compliance and long-term stakeholder engagement?	When communities are actively involved in decisions to reopen or extend closures, participatory processes are likely to foster higher compliance, cooperation and sustained engagement. Inclusive decision-making enhances perceived legitimacy and fairness, aligning management rules with local knowledge and priorities. In contrast, top-down decisions that limit community input are expected to reduce compliance and weaken long-term stakeholder engagement, even if ecological goals are achieved.	Governance process: participation level; decision-making structure Temporal flexibility: opening/closing rules; access ratio adjustment Activity (key mediating variable): effort and gear rules shaping compliance responses Additional mediating variables: institutional trust; enforcement capacity; communication quality
Under what ecological and social conditions do time-bound and cyclical closures generate tangible benefits that enhance fisher compliance, governance legitimacy and long-term support?	Time-bound and cyclical closures that align with ecological cycles and community harvesting rhythms are expected to deliver greater benefits, enhancing fisher compliance, perceptions of legitimacy and long-term support, whereas misaligned closures are less likely to generate these social outcomes. For example, closures that allow openings according to the best market conditions will render higher benefits and support.	Temporal alignment: duration; cyclicity; access ratio Ecological conditions: productivity; variability Market dynamics: price conditions; timing incentives Activity (key mediating variable): gear use; effort intensity; targeting behavior Social outcomes: compliance; legitimacy; support Additional mediating variables: governance capacity; enforcement strength; spatial visibility of benefits
c. Uncertainty and learning		
Do time-bound and cyclical marine closures increase resource users’ awareness of environmental variability and stock conditions?	Time-bound and cyclical closures, by requiring repeated engagement with management decisions and adaptive timing of access, encourage resource users to monitor environmental fluctuations and stock conditions more closely. Users participating in cyclical or temporary management systems are predicted to be more responsive to ecological signals, more aware of governance challenges and more likely to comply with rules compared to those in permanent or non-cyclical closures, where engagement is less frequent and feedback loops are weaker.	Temporal structure: cyclicity; duration; access ratio Feedback structure: frequency of closures/openings Activity (key mediating variable): fishing effort; monitoring behavior Spatial variability: stock distribution Additional mediating variables: governance responsiveness; environmental variability
Does the adaptive flexibility of time-bound marine closures enhance social-ecological resilience to environmental and social change?	Time-bound closures enhance resilience by allowing the periodic reassessment and adjustment of management rules in response to ecological and social changes. Systems with flexible, adaptive closure durations are expected to better maintain ecological sustainability, support community livelihoods and respond to shocks, compared to non-time-bound closures, which may be less responsive to changing conditions.	Temporal flexibility: adjustability of duration; cyclicity; access ratio Ecological variability Activity (key mediating variable): adaptive effort behavior; gear adjustments Spatial dynamics: redistribution of effort Additional mediating variables: institutional adaptability; climate shocks; market volatility
Can time-bound closures act as experimental or transitional measures that facilitate the adoption of broader or longer-term marine management strategies?	Time-bound closures provide a flexible, low-risk platform for testing management strategies. When these closures demonstrate ecological and social benefits – such as improved stock recovery or positive community engagement – they increase the likelihood of adoption of longer-term or more extensive management measures. Therefore, successful implementation and outcomes of time-bound closures are predicted to serve as steppingstones that inform and support the design of subsequent, more permanent management interventions.	Temporal design: duration; cyclicity; access ratio Experimental structure: trial design; phased implementation Activity (key mediating variable): regulated effort and gear constraints shaping experimentation outcomes Spatial scope: coverage of management experiment Additional mediating variables: learning mechanisms; governance feedback loops; institutional capacity
How do observed ecological, economic and social outcomes, as well as community perceptions of success, influence learning and the future design of marine closures?	Perceptions of success by local communities will play a stronger role in shaping future closure design and management decisions than objectively observed ecological or economic outcomes. Closures perceived as beneficial – through visible resource recovery, equitable access or tangible community benefits – are more likely to inform adaptive redesign and continued compliance, whereas closures that achieve ecological or economic goals but are not recognized as successful by users may have limited influence on future management choices.	Perceived outcomes: ecological; economic; social perceptions Temporal design: duration; cyclicity Activity (key mediating variable): fishing outcomes linked to effort and gear use Spatial visibility: distribution of benefits Additional mediating variables: communication channels; governance trust; enforcement credibility
d. Economic resilience		
How do time-bound *versus* permanent marine closures influence the intergenerational distribution of ecological and economic benefits under current and climate change conditions?	Under current environmental conditions, permanent closures are expected to provide higher benefits to future generations by ensuring long-term protection of resources. However, when considering climate change and environmental variability, time-bound closures that allow adaptive management may achieve higher long-term benefits by enabling adjustments to shifting ecological conditions. Therefore, the fairness of intergenerational cost and benefit distribution depends on both closure duration and the capacity for adaptive management in response to environmental change.	Temporal design: duration (permanent *vs.* time-bound); cyclicity Climate variability Activity (key mediating variable): effort allocation; gear use; targeting strategies Spatial persistence: resource distribution over time Additional mediating variables: governance enforcement; market structure; ecological productivity
How does the timing and predictability of harvest opportunities influence fishers’ economic resilience?	Scheduled and predictable harvests enhance fishers’ economic resilience by allowing them to align fishing effort with favorable market conditions, optimize income and plan livelihoods around resource availability. In contrast, unpredictable or *ad hoc* access may limit economic opportunities, increase risk and reduce overall resilience, even if ecological outcomes are positive.	Temporal predictability: cyclicity; access ratio Market timing: price cycles; demand patterns Activity (key mediating variable): effort allocation; gear use Spatial access: distribution of fishing opportunities Additional mediating variables: fuel costs; institutional constraints; market volatility
Under what design conditions do time-bound and cyclical closures support both economic benefits for fishers and long-term management outcomes?	Time-bound and cyclical closures will achieve a balance between economic benefits and management goals when they are aligned with the ecological traits of target species – such as reproductive cycles, mobility and vulnerability – and when fishing during open periods is carefully managed to minimize broader ecosystem impacts. Closures that match species’ biological rhythms and enforce sustainable harvest practices are predicted to allow stock recovery while providing predictable economic returns, whereas misaligned or poorly regulated closures may compromise either management or economic objectives.	Temporal alignment: cyclicity; duration; access ratio Species traits: reproductive cycles; mobility; vulnerability Activity (key mediating variable): harvest strategies; gear restrictions; effort control Spatial structure: habitat overlap Additional mediating variables: enforcement capacity; market incentives; ecological productivity
Can rotational (cyclical) closures be designed to optimize both ecological recovery and the economic well-being of fishing communities?	Rotational closures that are timed to align with ecological recovery periods and favorable market conditions can simultaneously enhance fish stock resilience and improve economic outcomes for fishers. By distributing access spatially and temporally, rotational closures may also reduce inequities between communities, ensuring more equitable opportunities to benefit from harvesting while maintaining long-term ecosystem health. In contrast, poorly timed or uniform closures may either compromise ecological recovery or fail to provide meaningful economic benefits, limiting both sustainability and community support.	Temporal structure: rotation schedule; cyclicity, access ratio Spatial structure: rotation pattern across areas Activity (key mediating variable): redistribution of effort across space and time Ecological outcomes: recovery rates Additional mediating variables: enforcement capacity; habitat heterogeneity; market incentives

## Ecological and fisheries sustainability

Most research on ecological outcomes has focused on permanent closures, showing that large non-time-bound closures can lead to strong conservation benefits, while small closures in networks can lead to conservation and fisheries benefits (*e.g.*, Green et al., 2009; Gaines et al., 2010; Sala et al., 2021). However, studies of time-bound or cyclical closures have primarily emphasized their role as fisheries management tools. A substantial body of work demonstrates that periodically harvested closures can provide fisheries benefits, particularly for fast-growing or moderately vulnerable species, by increasing biomass before harvest or improving catch efficiency (Cohen and Foale, 2013; Goetze et al., 2016, 2018; Carvalho et al., 2019a; Keith et al., 2020). However, evidence that these systems consistently deliver long-term biodiversity conservation outcomes remains limited, especially for highly vulnerable or long-lived species, and there is concern of overharvest events during open periods (Goetze et al., 2016, 2018).

Despite these concerns, and the limited empirical evidence supporting the effectiveness of temporary closures in achieving management goals, emerging research suggests that time-bound closures can contribute to ecologically meaningful outcomes and may serve as valuable complements to other management tools, including permanent MPAs. For example, studies at Isla Natividad, Mexico, where a fish refuge was designed to expire after a fixed period, found that protection helped maintain the genetic diversity of exploited fish populations and may enhance resilience to environmental stressors (Micheli et al., 2012; Munguía-Vega et al., 2015; Smith et al., 2022). Although these studies do not explicitly label the site as a temporary closure, it operates under a time-bound management regime (Villaseñor-Derbez et al., 2019), illustrating how temporal design can shape ecological outcomes. Similarly, fishing cooperatives in the Mexican Caribbean have implemented time-bound closures to protect Nassau grouper (*Epinephelus striatus*) spawning sites (Fulton et al., 2018). These examples indicate that, although management outcomes from temporary closures are not as thoroughly documented as those from permanent MPAs, the potential for carefully timed closures to simultaneously support fisheries and ecological objectives represents a fruitful avenue for research.

Management strategies can incorporate both non-time-bound and cyclical closures, using flexibility to balance management goals with socio-cultural needs. Cyclical, time-bound closures, for example, can be timed to biological rhythms – such as spawning periods – allowing sustainable harvesting while supporting management outcomes. Most studies have focused on species-specific recovery, showing that such closures can enhance stock biomass, abundance and fishery yields across diverse life histories, fishing scenarios and spatio-temporal patterns (Game et al., 2009; Carvalho et al., 2019b; Chen and Hastings, 2023). While non-time-bound MPAs can also protect highly vulnerable or slow-recovering species (Gnanalingam and Hepburn, 2015), specific access ratios in temporal closures can protect short-lived species during critical reproductive periods (Cohen and Foale, 2013). Yet, the ecosystem-level and trophic effects of time-bound or cyclical management remain largely underexplored (Table 1). The access ratio influences not only target stock recovery but also habitat resilience, predator–prey interactions and bycatch survival. Periodic human access can alter top-down trophic dynamics, and the use of unselective gear or pulse fishing may disproportionately affect slow-recovering species (Goetze et al., 2016). Considering gear selectivity, habitat sensitivity and species life histories can help identify thresholds where temporary closures provide broader ecological benefits while balancing fisheries objectives.

Future research should also explore how the synchronization of access regimes with ecological conditions influences fisheries outcomes and fisher behavior. MPAs can support stock recovery, but benefits depend on species mobility and larval dispersal (Planes et al., 2009). These challenges in observing benefits, can limit community support. In contrast, time-bound closures offer an alternative for low-mobility species, enabling stock buildup during closures and immediate harvest upon reopening. These visible gains could enhance community benefits and long-term engagement (Table 1).

## Governance, equity and participation

Balancing effective marine management with community engagement remains a central challenge. Non-time-bound closures are often favored for their simplicity – requiring primarily spatial decisions on size and location – which facilitates monitoring and enforcement (Smallhorn-West et al., 2020). While sometimes implemented through co-management, these closures often prioritize biodiversity conservation over sustained local use (Day et al., 2019). When not designed with a community-centered perspective, they can suffer from low community buy-in (De Santo et al., 2011; Bennett and Dearden, 2014). Moreover, their spatial boundaries and regulations are often designed to persist over long time horizons, which may not always align with the evolving needs, values and livelihoods of coastal communities, even though many non-time-bound MPAs undergo periodic management reviews (McClanahan et al., 2006).

Time-bound and cyclical closures could offer a more temporally responsive framework aligning the rhythms of human and non-human systems in settings where permanent MPAs face significant challenges. These approaches allow for periodic harvest and reassessment, and have the potential to foster deeper stakeholder engagement (Cinner et al., 2019; Quintana et al., 2021).

For example, in French Polynesia, *rāhui* are reemerging across the archipelagos and demonstrate strong adaptive potential. Traditionally, *rāhui* involved time-bound harvesting bans (usually a few years) imposed by chiefs who controlled defined coastal territories that extended into marine zones (Bambridge et al., 2019). Today, these closures take more diverse forms and are appealing to fishers since they lower the stakes of the initial decision to participate. Unlike long-term closures that are typically designed to persist for extended periods (although many are periodically reviewed), time-bound systems like many rāhui allow communities to “try out” management measures without forfeiting the possibility of future access (Fabre et al., 2021). This feature aligns with diffusion of innovations theory, which posits that practices that can be tested on a limited basis (“trialability”) are more likely to be adopted because users can assess their benefits and risks before committing to long-term change, an idea increasingly used to explain the spread of conservation initiatives (Mascia and Mills, 2018) Similarly, in Mexico, time-bound closures have been shown to align with local ecological knowledge and are typically established through collaboration between communities and relevant authorities (Quintana et al., 2021; Quintana and Basurto, 2021). The flexibility to revisit and adjust management decisions seems to offer a practical governance advantage in that stakeholder participation is required, which can reinforce stewardship among fishers by fostering a sense of ownership (Quintana and Basurto, 2021).

Future research could help reveal the unique adaptive governance dynamics of time-bound closures and the differences between cyclical and non-cyclical schemes with varying access ratios. In both French Polynesia and Mexico, the periodic decision points – whether to open a *rāhui* or renew a fish refuge – appear to create spaces where stakeholders frequently encounter and must negotiate one another’s knowledge, priorities and uncertainties. These are not just moments of administrative review; they instead seem to encourage platforms for adaptive governance, where the rules of engagement, who decides, who benefits and on what grounds, are not fixed, but actively shaped through practice (Quintana et al., 2021). More research could elucidate the extent to which and under what configurations time-bound and cyclical closures spawn new forms of social coordination. Rather than entrenching a static management regime, these systems are intrinsically responsive, enabling a kind of governance that adapts over time through stakeholder engagement and governance feedback.

## Uncertainty, learning and adaptive management

Uncertainty is a defining challenge in marine resource management, underscoring the need for strategies that can mitigate risks while remaining responsive to changing ecological and social conditions. Non-time-bound closures embody a precautionary approach, offering insurance against environmental shocks and prioritizing long-term population recovery when regeneration timelines are uncertain – an attribute that is critical for biodiversity conservation (Hopkins et al., 2020). In contrast, temporal closures enable more timely reassessment and adjustment. While reassessment is also possible within permanent MPAs, their fixed nature may constrain the integration of emerging ecological information or limit responsiveness to changing conditions.

Temporary closures, including time-bound and cyclical closures, offer a dynamic management framework with the potential to support observation, adjustment and learning – key elements for managing uncertainty in complex marine systems. By allowing periodic reopening of protected areas, these approaches create opportunities for stakeholders to observe ecological responses, evaluate management outcomes and iteratively refine management strategies. Because reopening periods are anticipated, fishers may become more engaged in monitoring ecological signals and assessing the results of protection, while scientists, managers and fishers can exchange observations and interpretations during these cycles. Studies of temporary closures in small-scale fisheries suggest that these iterative processes can foster participation, strengthen compliance and generate feedbacks that shape subsequent management decisions (*e.g.*, Oliver et al., 2015; Jupiter et al., 2017; Cinner et al., 2019). In some cases, the outcomes of temporary closures have prompted communities to rethink and modify their management arrangements. For instance, results from a multi-year closure in Mexico led local fishers to expand the protected area and extend the duration of protection (Quintana et al., 2021; Quintana and Basurto, 2021). Similar adaptive responses have been observed elsewhere: in both French Polynesia and Madagascar, initial closure arrangements evolved as fishers witnessed tangible benefits. In some cases, *rāhui* remained in place longer than originally planned, and fishers from Madagascar who had previously been skeptical came to support spatial restrictions after experiencing the positive effects of short-term octopus closures (Oliver et al., 2015; Fabre et al., 2021). While these cases illustrate how temporary closures can facilitate experiential learning and build support for management interventions, it remains unclear how consistently such adaptive processes emerge across different socio-ecological contexts or whether they translate into more effective management outcomes at broader scales. It is also important to note that closure periods are not strict statistical “controls”: income and other benefits from open periods can influence behavior and outcomes during subsequent closures, creating temporal spillovers that shape ecological and social dynamics. Further comparative research is therefore needed to determine when and under what conditions time-bound closures foster institutional learning and adaptive redesign. Further comparative research is therefore needed to determine when and under what conditions time-bound closures foster institutional learning and adaptive redesign. In particular, future studies should examine how the timing and repetition of these closures shape learning processes and adaptive capacity, which are critical for managing ecological and social uncertainty.

## Economic resilience

Time-bound and cyclical closures introduce a distinct economic dimension that warrants deeper investigation. A key area for future research is the temporal distribution of costs and benefits. While permanent, non-time-bound closures can generate substantial ecological and economic benefits, including increased fish stocks, catch volumes and tourism opportunities (Costello et al., 2024), time-bound and cyclical closures offer additional flexibility by allowing scheduled harvesting. This scheduling can help communities balance resource use across short-term livelihood needs and longer-term management goals, supporting more equitable intergenerational trade-offs in contexts where local livelihoods depend on predictable access to resources (Oliver et al., 2015; Silas et al., 2022).

Research is needed to explore how such closures can alleviate the financial burdens often associated with marine conservation by offering predictable, high-value harvest opportunities. These mechanisms may serve as economic buffers, functioning similarly to savings accounts – allowing fishers to “store” biomass and extract it when most needed or when market conditions are favorable. The case of Baja California, where cooperatives drew on sea urchin and abalone reserves to offset pandemic-related losses, illustrates the potential for time-bound strategies to enhance economic resilience (Villaseñor-Derbez et al., 2023).

Future studies should examine how these temporal dynamics influence fishers’ willingness to comply, the sustainability of harvest cycles and the broader economic feasibility of cyclical closures in variable market and ecological conditions.

## Conclusion

Non-time-bound closures, such as traditional MPAs, treat permanence as the only effective structuring of time, overlooking the diversity of marine management strategies worldwide. Understanding how temporal factors, duration and cyclicity, interact with spatial design is essential for effective fisheries management. Our framework highlights these temporal dynamics, offering new directions for research on adaptive management. As climate change and other pressures intensify, flexible, time-bound strategies may become increasingly important for supporting sustainable fisheries and resilient socio-ecological systems. A clearer temporal typology of closures can reveal which designs perform best under different ecological and social conditions, enabling more context-sensitive management and research.

## Data Availability

Data sharing not applicable to this article as no datasets were generated or analyzed during the current study.

## Long descriptions

### Table 1. Long description

Major research areas and associated questions examining the ecological, social, governance, and economic dimensions of area-based marine management through a temporal lens, explicitly incorporating activity (e.g., gear, effort and targeting behavior) and additional mediating variables (e.g., governance capacity, market dynamics and ecological heterogeneity) that condition outcomes across systems.

Navigate back to Table 1..

### Figure 1. Long description

A hierarchical flowchart begins with a top central question box: Does the closure system expire?

Two paths branch downward from this question:

1. The left path, labeled No, leads to a dark blue box: Non-time bound intended to be permanent. Below this, a secondary question box asks: Will fishing be allowed in this closure intermittently?

- A Yes branch leads to a teal box labeled Cyclical.

- A No branch leads to a teal box labeled Non-cyclical.

2. The right path, labeled Yes, leads to a dark blue box: Time bound has expiration date. Below this, the same secondary question box asks: Will fishing be allowed in this closure intermittently?

- A Yes branch leads to a teal box labeled Cyclical.

- A No branch leads to a teal box labeled Non-cyclical.

Navigate back to Figure 1..

### Figure 2. Long description

The diagram is organized into two main sections.

Top section, Panel A: A red square wave graph plotted against a horizontal arrow labeled Time. The low state of the wave is labeled Closure and the high state is labeled Access. A horizontal bracket below one full cycle of the wave (one high and one low segment) is labeled Period, T.

Bottom section, Panels B through D: Three side-by-side square wave graphs showing different duty cycles.

- The left graph is labeled 30 percent and shows narrow peaks with wide troughs, indicating short access periods relative to closures.

- The middle graph is labeled 60 percent and shows peaks that are slightly wider than the troughs, indicating access periods that exceed closure periods.

- The right graph is labeled 90 percent and shows very wide peaks with extremely narrow troughs, indicating nearly continuous access with very brief closure intervals.

Navigate back to Figure 2..

### Figure 3. Long description

The image consists of two panels, B and C, showing a red line that steps between an upper Access level and a lower Closure level.

Panel B, titled Rahui Papara, shows a long initial Access period. The line then drops to the Closure level for a duration labeled 2020 4 Y. It rises briefly to the Access level for a duration labeled 2024 1 Day, before dropping back to the Closure level for a duration labeled 2024 3 Y.

Panel C, titled Octopus Closure Madagascar, shows a repeating cyclical pattern. The line starts at the Access level, then drops to the Closure level for 3 M in 2015. It rises back to Access for 9 M O. This cycle repeats twice more, with 3 M closures in 2016 and 2017, each followed by a 9 M O access period. The horizontal segments for access and closure in this panel are of uniform length relative to each other.

Navigate back to Figure 3..
