# Supplementary material for: Temporal dimensions in marine spatial closures
Source: Camb Prism Coast Futur. 2026 Jun 8;4:e18. doi: 10.1017/cft.2026.10034 (PMC13375839; doi:10.1017/cft.2026.10034)
Supplement: Peer review history

# Reviewer report: Review: Temporal dimensions in marine spatial closures — R0/PR2

**DOI:** 10.1017/cft.2026.10034.pr2

Summary

The paper calls for a greater focus on temporal management in marine conservation by introducing a framework for understanding temporary marine closures through three temporal dimensions: duration, cyclicity, and access ratio. It is very well written, and I agree that temporary closures can be very useful and important for fisheries management. There is also a clear gap in research on their effectiveness and role in broader conservation strategies and the framework presented here does have the potential to help address this.

However, the current framing creates a false dichotomy between temporary closures and permanent no-take areas, when they are in fact complementary tools already being used across the world for different primary objectives—conservation of biodiversity and fisheries management. By presenting temporal closures as a replacement for MPAs, the manuscript comes across as biased, generally highlighting only the positives of temporal closures and the negatives of MPAs. This issue is exacerbated by poor referencing, with many claims lacking any supporting evidence.

However, I believe most of these issues can be fixed relatively easily and the manuscript would be much stronger if it presented temporal closures as a complementary tool within the broader marine management toolkit. I see no reason why temporal closures can’t be one of the many strategies an MPA can offer—and they are already being used in conjunction across the world. Additionally, the unsupported claims could be reframed as research gaps that the manuscript aims to highlight and encourage further work on. With these adjustments, the paper could become a valuable contribution to the field.

Major

I recommend avoiding the false dichotomy of temporary closures versus no-take areas. We should encourage a combination of both. While very large permanent closures can be challenging and inequitable, smaller no-take areas—especially when forming networks—offer significant benefits. Therefore, I would avoid statements suggesting they are rarely feasible (e.g., lines 4–8). There is substantial evidence that networks of well-designed, fully protected areas can provide fisheries and nutritional benefits to communities. I would avoid claims that they cannot work where people rely on marine resources (e.g., in the abstract and discussion).

I agree that temporary closures receive less attention and research, so focusing on this gap is valuable. However, arguing for one approach over the other is problematic because temporary closures will never match the long-term biodiversity conservation benefits of permanent closures. Temporary closures are very useful as a fisheries management tool and should be presented as complementary rather than competing. There benefits for conservation are not as well understood and this should be the research gap that is pointed out.

Paragraph 66–74: Either clarify that you are referring only to very large fully protected areas (with a definition or example) or, as suggested above, emphasize how common temporal closures are globally yet remain poorly understood.

Introduction: I think the authors need to highlight that temporal closures are primarily used as a fisheries management tool. While you can discuss their conservation potential, they are widely used for fisheries management around the world, and this point is somewhat missing. Currently, the introduction emphasizes biodiversity conservation, but the body focuses on fisheries management, creating confusion and suggesting that relevant literature may have been overlooked.

Line 189–190: The statement about conservation benefits is not entirely accurate and needs references. Temporary closures tend to provide short-term fisheries benefits for less vulnerable (e.g., fast-growing) species, but evidence of long-term biodiversity conservation benefits is currently rare and poorly supported. Add a balanced, referenced statement acknowledging this. Some suggested references:

• https://doi.org/10.1111/1365-2664.13417

• https://doi.org/10.1111/1365-2664.13047

• https://doi.org/10.1016/j.biocon.2016.08.038

• https://doi.org/10.1016/j.marpol.2012.05.010

• https://journals.plos.org/plosone/article?id=10.1371/journal.pone.0240322

Section 187: The authors do not discuss negatives here, such as the tendency for overharvesting after closures. Again, a more balanced approach is needed.

Line 233–237: MPAs are rarely static, with most having management plans and zoning schemes that are periodically reviewed. I would avoid framing this as “one is flexible, the other is not.” Instead, note that temporal closures may allow for timelier reassessment, as reassessment is not absent from MPAs. Similarly, for line 262, say “more responsive” rather than implying MPAs are completely static. For lines 271–272, delete the sentence suggesting MPAs lack adaptive management, as they are critical for identifying ecological insights by providing areas relatively free of anthropogenic impacts.

Line 269–271:

How are temporary closures immune to these issues? Isn’t this the point of a precautionary approach—to provide insurance against environmental shocks? This should be framed as a positive rather than a negative.

Line 273–282:

Incorporate references or examples to support claims in this paragraph.

Lines 283–291:

Avoid using a single example to make broad claims (e.g., that time-bound closures “lead to more responsive and effective management over time”). Instead, present the example as promising case study, then highlight the research gap (“we don’t know if this applies elsewhere or at scale”), and then make the future research recommendation (as you do in lines 289–291).

Lines 296–299 and 302–304:

These statements lack evidence. Permanent closures can provide significant tourism benefits, so they are not necessarily a “sacrifice from current generations.” In fact, there is little evidence of economic costs, even for fisheries, and more evidence of benefits. A more balanced and referenced approach is needed. Suggested reference:

• https://doi.org/10.3989/scimar.05417.080

Line 314–315: I don’t think temporary closures have a primary goal of biodiversity conservation—but they are being used extensively for fisheries management. If you want to argue for inclusion into the 30 x 30, you will need to provide concrete evidence of biodiversity conservation targets and benefits, so I think the distinction made by the 30x30 initiative is understandable. Why not reference fisheries-related targets that align better with your argument, such as Target 5 (Sustainable Harvesting of Wild Species) or Target 10 (Sustainable Fisheries Management)?

Minor

Instead of permanent closures I recommend referencing the MPA Guide here and using their proposed terminology throughout. For example, the sentence at Line 57–60: could use “fully protected areas.” https://www.science.org/doi/abs/10.1126/science.abf0861

Line 60: I suggest adding another reference here, given that you state “several” at the start of the sentence.

Line 70: You use the term “scholars” but only cite one paper without presenting a counterargument. There is now substantial evidence that MPAs provide spillover, particularly larval spillover, which can offset displacement (e.g., https://www.science.org/doi/full/10.1126/sciadv.adt0216). Another important point is that temporal closures also suffer from displacement issues, potentially without the benefit of spillover to offset this, as they are rarely in place long enough to build up large, old, fecund individuals.

Paragraph 101–111: I recommend revising this paragraph to discuss broader regions rather than just two countries, as periodic closures are used widely. Two useful papers for this are below; the Carvalho paper includes a helpful map:

https://doi.org/10.1111/1365-2664.13417

https://doi.org/10.1111/1365-2664.13047

Line 161: Use “well-timed closures” rather than “openings.”

Line 193: Follow-up studies in 2012? Were these not permanent reserves? Also, evidence of resilience remains somewhat inconclusive, even for fully protected areas, so one study is insufficient to support this statement. I recommend removing this altogether, as it is not essential for the purpose of this study.

Line 198: I don’t see why this needs to be framed as one or the other. I suggest changing this to emphasize the need for flexibility in using both permanent and cyclical closures as part of the management toolkit. As you note, cyclical closures allow alignment with biological rhythms, and the example of a spawning aggregation above is a good illustration.

Line 203–204: Change “best suited” to “can also protect,” as they protect broader biodiversity and thus provide benefits to both.

Line 210: This needs a reference, e.g. https://doi.org/10.1016/j.biocon.2016.08.038

Line 216–220: I recommend removing the time argument here, as time-bound closures face the same issue. However, the “visibility” of benefits is a very valid point, and you could even add that this visibility might help build support for permanent closures, as fishers can see the buildup of fish during a harvest.

Figure 1: I’m not sure what this figure is demonstrating. Are readers expected to be familiar with what is happening in each country?

# Reviewer report: Review: Temporal dimensions in marine spatial closures — R0/PR3

**DOI:** 10.1017/cft.2026.10034.pr3

This article represents an important contribution to the literature and is very close to ready for publication. I have several content-related comments, several other minor suggestions (e.g., word-smithing and idea flow, Table and Figure suggestions, and a few typos).

Content-related comments:

- In my work with area-based fisheries management I am familiar with a tripartite framework with three dimensions: space, time, and activity. Your article focuses in on the distinctions between space and time, given that space dimension has been more heavily emphasized compared to the time dimension. But it seems that the activity dimension is almost completely sidelined in the framing of the article, with choice of gear mentioned as a sidenote related to “how access is managed.” It is completely fine and important that your article focuses on bringing the time dimension more fully to light with respect to space, but it would help to:

o A. More fully position your work in the larger conversation to explicitly acknowledge that “activity” has been the most widely studied, with “space-time” a more recent area of focus… and that your work specifically targets the most under-investigated aspect: time.

o B. Explicitly acknowledge the complexity of the activity dimension as a factor that needs to be controlled for in research on temporary closures, to support teasing out the role of the time dimension from the intertwined dimensions of space and activity. You have started to acknowledge this complexity in your discussion of gear choices on Lines 159-164 and 207-213, but more directly integrating the three dimensions of space, time and activity in the framing of your article and in the proposed research questions would help make this framework more useful in application. As it currently stands, as a reader I am left thinking, “well, yes, it’s great to focus on time but there is infinite diversity of what kinds of gears might be used across contexts so it would be hard to discern any meaningful results without actively integrating the activity dimension in the framing of the research questions.”

- Another lingering question that would be good for you to explicitly is whether you are primarily dealing with closures that remain in the same space (potentially expanding/shifting but largely in the same location) or if your framework is inclusive of more dynamic ocean management in which the space would change alongside variable time constraints.

- To help clarify the above points, and strengthen the contribution of this framework, here are specific suggestions:

o Clearly position your work in relation to other typologies of ABMTs. You could use “Hilborn et al., 2022. Area-based management of blue water fisheries: Current knowledge and research needs. Fish and Fisheries, 23: 492-518.” You have already cited this article, but not in the sense of clarifying how your framework fits into and potentially helps to augment / refine this larger framework of ABMT types. Perhaps you could even suggest a revised version of the overall Hilborn typology (p. 499) that would more effectively integrate the axes you are teasing out in this article?

o Add a column in Table 1 that identifies specific variables of interest for each research question, including dimensions of activity and space where relevant. For example, the second research question under Fisheries Sustainability could list the following variables of interest: [Activity (gear selection), Habitat sensitivity, Species life histories]

o Consider whether activity needs to be incorporated in most/all of the research questions as a confounding variable. And consider adding a specific example of how you would integrate the activity dimension, e.g., how would you build the influence of varied activities into research on designing a best-fit access ratio? Or e.g., to operationalize the 5th question under Governance, Equity and Participation (Under what ecological and social conditions do time-bound and cyclical closures generate tangible benefits that enhance fisher compliance, governance legitimacy, and long-term support?). And perhaps there needs to be a research question to IDENTIFY additional mediating variables that play similar roles to the activity dimension that would also need to be taken into account to truly separate out the influence of time-bound and cyclical marine closures?

Wording, idea flow,

- Lines 12-15: It seems that there are really only two temporal dimensions you identify (axes of duration and cyclicity) and that access ratio is an element of cyclicity. Should you revise the abstract to rephrase the following sentence to remove the suggestion that there are three distinct temporal dimensions? Relevant excerpt: “We introduce a framework that distinguishes three key temporal dimensions—duration, cyclicity, and access ratio—to clarify how different temporal designs shape not only ecological, but also social, and governance.”

- Impact Statement (Lines 37-49): You note this elsewhere in the article, but I suggest you consider including a statement in the impact statement section that fisheries management approaches which are integrated with social relations, cultural traditions, and local governance systems are more likely to be sustainable over a longer-term…

- Lines 87-89: Suggestion for clarifying language: “This limitation is also present in discussions around Other Effective Area-based Conservation Measures...” Or maybe limitation is the wrong word... perhaps default? or tendency? (Here is the original excerpt: “This includes Other Effective Area-based Conservation Measures (OECMs) which must be “ongoing and for the long-term” (IUCN WCPA Task Force on OECMs, 2019).”)

- New and reordered paragraph (Lines 91-100): First, I suggest a new paragraph begin at “Yet, temporal management… (Line 91).” Second, the flow of ideas in the next few sentences could be improved. I offer you a suggested revised paragraph, with a few notes afterward: “Yet, temporal management is neither new nor marginal: in the Global North, fisheries have long used seasonal closures, rotational harvests, and real-time restrictions; in the Global South, communities have long practiced non-permanent closures rooted in local stewardship traditions, representing a rich but underexplored dimension of temporary marine management. Research on these tools has primarily focused on ecological outcomes, examining both their benefits and potential downsides (e.g., Hart...). However, diverse examples of current temporal fisheries management around the world highlight the importance of studying not only ecological outcomes, but also the socio-cultural and political dimensions of temporary marine conservation.”

o Note 1: Suggested moving “research on these tools” down after the subsequent sentence “In many regions... underexplored dimension of temporary marine management.”

o Note 2: The way it was written originally, it seemed like you’re saying research on ecological outcomes has primarily focused on the application of these tools in the Global North... but then in subsequent paragraphs you cite lots of research on outcomes of temporary closures in the Pacific Islands, Indian Ocean, and Mexico... in my suggested re-write of the flow of this paragraph I just said “research has focused on ecological outcomes,” and the reordering removes the suggestion that that is mainly the case in the Global North. If you agree, you may want to update the list of citations that follows “Hart et al.” to ensure it includes examples of ecological research from both Global North and Global South.

o Note 3: In my suggested revision I included “socio-cultural and political dimensions” in the final sentence – does that fit, or would you like to keep “social and cultural”?)

Typos:

- Lines 127-128: In the sentence, “This broad categorization obscures how temporal design shapes not only ecological, but also governance, social outcomes,” should the end say “governance and social outcomes”? Something seems missing and I think the comma is a typo.

- Recurring: Do you want to use the Roman Numeral “I” instead of “1” when referring to Table 1? You have used “Table I” throughout the text, but in the Table itself uses “1”.

- Figure 3 typo: In the visual portion of Figure, 3, it appears that 2019 (3Y) is repeated in the Papara portion of the figure and I am wondering if the second one should say “2021: 3Y” or "2022: 3Y' instead? (But 2019 to 2024 doesn’t allow for two distinct periods of 3 years, so I am confused...)

Figure / Table Suggestions:

- Figure 1 / Access Ratios: I included a comment above suggesting that you may want to update the abstract to refer only to two temporal dimensions, since access ratio appears to be a subsidiary concept. This is my understanding because the caption of Figure 1 clearly states that the classification you are proposing is based on TWO dimensions: System duration and Cyclicity, and that access ratio is a sub-attribute of these larger dimensions. If that is NOT the case – if you envision access ratio as a third temporal dimension (as you stated in the abstract), then you would instead want to update the caption here and clarify how all three dimensions interact at a high level. (Note: You have also listed only two dimensions in the introductory statement for Future research directions – Line 181, and in the Conclusion – Line 317.

- Table 1: I suggest breaking Table 1 into four distinct tables with research questions for each area of research. This would allow you to eliminate the first column of the table that is currently blank much of the time. It would also allow for easier placement of the Tables in the article. (Also, referring back to the Typo list – do you want to refer to Table 1 as “Table I” or “Table 1”? If you end up adding more tables as suggested, would you refer to them with roman numerals or numbers?)

# Reviewer report: Review: Temporal dimensions in marine spatial closures — R0/PR4

**DOI:** 10.1017/cft.2026.10034.pr4

**Authors:** Matthew Clark

**Affiliations:** University of Sydney CAR, Australia

Matt Clark; University of Sydney; Thriving Oceans Hub.

This is a really nice paper and I thank the authors and editor(s) for the opportunity to review. The authors formally define three important aspects of temporary marine closures—duration, cyclicity, and access ratio—to refine our language and thinking about marine conservation. I agree with the authors that refining our thinking and lexicon will enable both better comparative studies. I would even expand this to argue that the ideas presented in this paper can also enable better development of formal theory, both in analytic and computational modelling. My main critique of the paper is that it would be nice to see some reflection on this from the authors. I can imagine a small sub-discipline of purely theoretical papers looking at how these various aspects relate to social and ecological dynamics and associated outcomes. I would really appreciate the authors’ thoughts on what this corpus might look like.

My only other substantial critique, which is really not all that substantial, is that I don’t buy the notion that access ratio only applies to cyclical closures. I give more detail below referencing the lines where it appears in the text. This comment also has bearing on figure 1.

Again, this is a nice paper and I would like to see it published after some slight modifications and further reflections on how better defining these concepts can help us build better theory in conservation; something that is much needed in my opinion, as we escape the trappings of being a “crisis discipline.”

There are two sets of line numbers on the document; I am referencing the numbers in blue

Line 15: “Implemented” might be better here as “flourishing” is a bit value laden.

Line 38: This is more stylistic, but I don’t really see the clause after “yet” in this line to be at odds with the first.

Line 53: “MSP” is an unnecessary acronym to introduce as it is never used again in the manuscript. Suggest removing “(MSP)”.

Lines 71-74: I know this sentence is intended to capture broad strokes, but paper parks are an issue in Global North countries too. For example, there is very little enforcement across Australia’s entire MPA network, including in the Great Barrier Reef.

Line 75: I wouldn’t say this is just state-imposed MPAs. Conservation projects run by NGOs have historically had lots of issues with elite capture and community-led programs can have issues with gender representation (e.g., men running the program make the closure in areas where women are more likely to go).

Line 87: You might mention that in conservation, the impermanence/discontinuation of protections is becoming a “hot topic” of study. E.g. https://www.nature.com/articles/s41559-025-02910-5

*This citation is self-referential; please do not feel obligated to include.

Line 87: Missing date in citation.

Line 92-93: You might mention the recently proposed mobile MPAs here - https://www.science.org/doi/full/10.1126/science.aaz9327

Line 124: This sentence makes it sound like you’re talking about the closures/areas themselves, rather than the temporal aspect(s) of closures vs the spatial aspect(s).

Line 140: I get that access ratio only applies to cyclical systems, but maybe good to just include access ratio at the end of this sentence too as to introduce all three concepts together (as in the abstract).

Line 155: This is a non-specific comment, so apologies if it’s annoying, but in Fiji there are cyclical rotational closures that are opened in the event of a hurricane to help people buffer the shock. I’m wondering if that still counts here given that you use the word “never”.

Line 159: I’m not convinced that access ratio only applies to cyclical systems. For event-triggered temporary closures there will still be an access ratio in practice, even if it may or may not be an intentional aspect of the closure system. Certainly in conducting comparative work (as this paper nicely sets up!), one could study the differing access ratios of various event-triggered closure systems. The period would be more difficult to define (as in fig 2), but the issue is not insurmountable; and as you say in the fig 2 caption “it does not require strictly repetitive patterns as those shown in these examples; the lengths of open and closed periods can vary dynamically across time frames, potentially reflecting adaptive management strategies.”

Line 245: Maybe not necessary to cite, but this aligns with a key insight from Diffusion of Innovations theory, which posits that “trialability” of innovations is more likely to lead to their uptake. There is even some work on DOI in conservation. See here: https://conbio.onlinelibrary.wiley.com/doi/full/10.1111/conl.12442

Line 273-282: Just something for the authors to think about: this paragraph positions open/closure periods as a sort of experimental approach where differences in times of opening and times of closure can be compared. But I would think that there are some temporal spillovers, where income from the open period is relied upon during the closure period, for example. Either here or elsewhere, I might caution against the comparison of open/closure periods as a sort of experiment where the impacts of closures can be assessed using the open periods as a control.

Figure 1: I find this figure a bit overly complicated/confusing. As mentioned above, I don’t think access ratio only applies to cyclical closures. If the authors agree, the figure will have to be redesigned anyway, which I think would be for the best.

# Editor report: Recommendation: Temporal dimensions in marine spatial closures — R0/PR5

**DOI:** 10.1017/cft.2026.10034.pr5

**Affiliations:** Wildlife Conservation Society, United States

General comments: I am in agreement with the reviewers that a revised version of the manuscript will add to the literature and discourse on area-based management with clear attention to the reviewers’ queries. Reviewer 1 recommends a reframing such that temporary closures are not set up against permanent no-take closures as an either/or dichotomy. I agree with their suggestion that permanent no-take closures and temporary closures are complementary tools being used for different primary objectives – though I would argue that temporary closures are not always used for purely fisheries management objectives. Reframing around a broader set of “management” objectives, instead of focusing as explicitly on “conservation” may help – to that end, I would suggest replacing the term “conservation” with “management” throughout. Revisions should also address Reviewer 2’s concerns about more attention to the activity dimension of area-based fisheries management and how this can vary greatly across different types of temporary closures.

Specific comments:

Lines 60-62: Note that there is no consensus on the nature of this 30% protection within the conservation community, with some groups advocating for fully or highly protected, while others maintain it is at the discretion of Parties to the CBD to make this determination.

Line 89: There is still a lot left for interpretation regarding what qualifies as “ongoing and for the long-term”. The authors should recognize that under some circumstances (e.g., non-time bound, cyclical, low access ratio) there may be a compelling case for counting biodiversity benefits of temporary closures against national and international commitments, but this will not necessarily hold for all temporary closures. A discussion of these differences and nuances is likely to be beyond the scope of this paper.

# Editor report: Decision: Temporal dimensions in marine spatial closures — R0/PR6

**DOI:** 10.1017/cft.2026.10034.pr6

**Affiliations:** Geography, Cambridge University, United Kingdom of Great Britain and Northern Ireland

No accompanying comment.

# Reviewer report: Review: Temporal dimensions in marine spatial closures — R1/PR8

**DOI:** 10.1017/cft.2026.10034.pr8

**Authors:** Matthew Clark

**Affiliations:** University of Sydney CAR, Australia

My remaining comments are mostly just about language:

Line 1 - I find it a bit weird to start the abstract by saying essentially “in theory large permanent closures are the best”, when the focus of this paper is on how we can use a better set of concepts to develop evidence and theory to help us do conservation better. It seems like “theory” in this opening sentence is being used in a colloquial sense rather than to mean formal scientific theory. But, then in the manuscript you present you briefly touch on the benefits of your framework for “theory” in a more formal sense. For example, this framework could be used to develop formal theory of how duration and cyclicity can be better timed with adjacent seasonal agricultural resources to smooth incomes in resource-dependent economies. This might be a very effective way to conserve marine biodiversity in practice. On this same note, it’s odd to say that something is viewed a certain way in theory. Are the authors referring to specific theoretical work on fishers' perceptions of biodiversity conservation?

In addition to the semantic bit, I actually don’t agree with this sentiment. Large permanent closures are increasingly viewed as a highly INEFFECTIVE strategy for some species/contexts. I think you could just as easily say that because large contiguous protected areas are difficult to monitor, high resolution, dynamic management is viewed as the most effective strategy for conserving biodiversity (in specific cases) (see: https://www.science.org/doi/10.1126/sciadv.aar3001).

Overall, I apologize for being long-winded but would just encourage the authors to think a bit more carefully about what they’re trying to say with this opening line of the paper.

Figure 1 - Maybe put “intended to be permanent” and “has expiration date” in parentheses to make clear that they are meant to be extra explanation of what’s meant by the “non-time bound” and “time bound” labels.

Also, it seems a little off to say that the closure is intended to be harvested. It’s the resources that are harvested and the closure is opened. Maybe the white boxes should say something like “is the closure ever opened?” or something similar.

# Reviewer report: Review: Temporal dimensions in marine spatial closures — R1/PR9

**DOI:** 10.1017/cft.2026.10034.pr9

Great work incorporating review comments -- the paper looks great! My only very minor thought is that you may want to double check whether there are any places in the text where the presentation of system duration, cyclicity, and access ratio continue to present them as three temporal dimensions rather than access ratio as a sub-element of cyclicity. (E.g, Lines 51. And the R1 version may include the old (original) abstract at the top, but the original list of three dimensions, so double check that the abstract is updated!

# Editor report: Recommendation: Temporal dimensions in marine spatial closures — R1/PR10

**DOI:** 10.1017/cft.2026.10034.pr10

**Affiliations:** Wildlife Conservation Society, United States

Overall, the queries to the original submission have been satisfactorily addressed. I would encourage the authors to make very minor final changes as per the reviewers' comments prior to final acceptance.

# Editor report: Decision: Temporal dimensions in marine spatial closures — R1/PR11

**DOI:** 10.1017/cft.2026.10034.pr11

**Affiliations:** Geography, Cambridge University, United Kingdom of Great Britain and Northern Ireland

No accompanying comment.

# Editor report: Recommendation: Temporal dimensions in marine spatial closures — R2/PR13

**DOI:** 10.1017/cft.2026.10034.pr13

**Affiliations:** Wildlife Conservation Society, United States

The authors have responded well to the additional quite minor comments from the two reviewers. I recommend moving this forward to publication.

# Editor report: Decision: Temporal dimensions in marine spatial closures — R2/PR14

**DOI:** 10.1017/cft.2026.10034.pr14

**Affiliations:** Geography, Cambridge University, United Kingdom of Great Britain and Northern Ireland

No accompanying comment.
